# Noxious stimulation in children receiving general anaesthesia evokes an increase in delta frequency brain activity

**DOI:** 10.1016/j.pain.2014.09.006

**Published:** 2014-11

**Authors:** Caroline Hartley, Ravi Poorun, Sezgi Goksan, Alan Worley, Stewart Boyd, Richard Rogers, Tariq Ali, Rebeccah Slater

**Affiliations:** a Department of Paediatrics, University of Oxford, Oxford, UK; b Nuffield Department of Clinical Neurosciences, University of Oxford, Oxford, UK; c Department of Clinical Neurophysiology, Great Ormond Street Hospital for Children, London, UK; d Nuffield Department of Anaesthesia, John Radcliffe Hospital, Oxford, UK

**Keywords:** EEG, Anaesthetics, Paediatrics

## Abstract

More than 235,000 children/year in the UK receive general anaesthesia, but it is unknown whether nociceptive stimuli alter cortical brain activity in anaesthetised children. Time-locked electroencephalogram (EEG) responses to experimental tactile stimuli, experimental noxious stimuli, and clinically required cannulation were examined in 51 children (ages 1–12 years) under sevoflurane monoanaesthesia. Based on a pilot study (n = 12), we hypothesised that noxious stimulation in children receiving sevoflurane monoanaesthesia would evoke an increase in delta activity. This was tested in an independent sample of children (n = 39), where a subset (n = 11) had topical local anaesthetic applied prior to stimulation. A novel method of time-locking the stimuli to the EEG recording was developed using an event detection interface and high-speed camera. Clinical cannulation evoked a significant increase (34.2 ± 8.3%) in delta activity (*P* = 0.042), without concomitant changes in heart rate or reflex withdrawal, which was not observed when local anaesthetic was applied (*P* = 0.30). Experimental tactile (*P* = 0.012) and noxious (*P* = 0.0099) stimulation also evoked significant increases in delta activity, but the magnitude of the response was graded with stimulus intensity, with the greatest increase evoked by cannulation. We demonstrate that experimental and clinically essential noxious procedures, undertaken in anaesthetised children, alter the pattern of EEG activity, that this response can be inhibited by local anaesthetic, and that this measure is more sensitive than other physiological indicators of nociception. This technique provides the possibility that sensitivity to noxious stimuli during anaesthesia could be investigated in other clinical populations.

## Summary

Clinically required noxious cannulation performed in children receiving sevoflurane monoanaesthesia causes a change in electrophysiological brain activity.

## 1 Introduction

The prevention of pain is one of the primary goals of anaesthesia during surgical procedures. While it is clear in both adults and children that anaesthesia can supress autonomic and motor responses following noxious stimuli, it is not known whether abolition of these measures equates to the provision of adequate analgesia [5]. Indeed, the concept of balanced anaesthesia [19] is based on our understanding that different compounds can independently affect analgesia, muscle relaxation, reduction or elimination of autonomic reflexes, and amnesia.

Cerebral cortical processing is a fundamental component of pain perception [33]. In nonverbal populations, measures of cortical activity may provide the best insight into whether an individual is in pain, and in the case of the anaesthetised patient, whether the anaesthetic provision is antinociceptive. In adults, recent investigations report that noxious stimuli administered during anaesthesia alter electroencephalography (EEG) activity. While many studies, in both animals [23], [24] and adult patients [3], [12], [16], [21], [31], report an increase in delta activity, a smaller number of studies also report the opposite finding [2], [12], [17], [31]. The observation that noxious stimulation in anaesthetised adults can lead to both increased and decreased delta activity has been attributed to the different doses and types of anaesthetics used in these studies, and to the different surgical procedures under investigation [2], [3], [26], [31]. As delta activity is the dominant pattern of brain activity in anaesthetised subjects, how this activity is modulated by external events, such as nociceptive stimulation, is important in understanding how the brain processes information when patients are in an unresponsive state.

In the UK, more than 235,000 children admitted to hospital each year receive an operation or investigation under general anaesthesia [9]. To date, cortical responses to noxious stimuli have not been investigated in anaesthetised children. As the brain continually undergoes development throughout childhood and adolescence, it cannot be assumed that children will respond to noxious stimuli in the same way as adults when anaesthetised. In the first few years of life there is a rapid increase in myelination [25] and an increase in synaptic density, followed by a period of synaptic pruning until mid-adolescence [14]. These developmental changes are reflected in changes in EEG frequency [11] and synchrony [35], and grey and white matter volume [25]. Additionally, there is a relative lack of research into the effect of drugs on children, and their effects at different ages. Recording direct measures of neurophysiological brain activity in response to noxious stimulation has the potential to provide a more complete understanding of how nociceptive information is being processed in the anaesthetised child. A first step toward this goal is to characterise the electrophysiological brain response evoked by a controlled nociceptive stimulus in children who are receiving a fixed dose of a single anaesthetic agent.

Due to its wide therapeutic index and fast speed of induction, sevoflurane is the preferred agent for gaseous anaesthetic induction in children. The study aim was to establish whether tactile (innocuous) and noxious stimuli evoke changes in electrophysiological brain activity of children receiving sevoflurane monoanaesthesia. A novel method of time-locking the EEG to clinical and experimental procedures was developed. Based on the literature in adults [3], [12], [16], [21], [31], which shows that noxious stimulation can evoke an increase in delta activity, and following a pilot study performed in 12 children, which showed results consistent with this observation, we hypothesised that noxious stimuli would cause an increase in delta activity. We aimed to test whether clinical cannulation and experimental innocuous and noxious stimuli evoked this response. In addition, we examined whether this increase in delta activity was blocked by the application of local anaesthetic.

## 2 Materials and methods

### 2.1 Subjects

Ethical approval was granted by the Oxford Research Ethics Committee of the National Research Ethics Service. Informed written parental consent and, where appropriate, the child’s assent, were obtained prior to each study. The study conformed to the standards set by the Declaration of Helsinki and Good Clinical Practice guidelines.

**Fig. 1:**
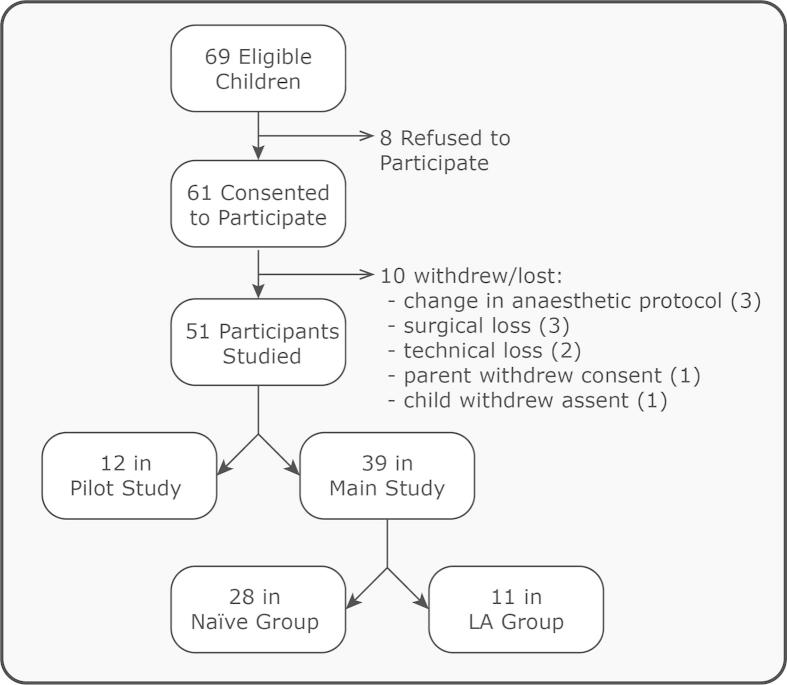
Recruitment flow chart. LA, local anaesthetic.

Fifty-one children (number of males = 26) aged 1–12 years receiving an elective operation or investigative magnetic resonance imaging (MRI) scan under general anaesthesia were recruited from the John Radcliffe Children’s Hospital, Oxford between July 2012 and February 2014 (see recruitment flow chart in Fig. 1). This age range was selected because an end-tidal concentration of 2.5% is equivalent to 1 minimum alveolar concentration of sevoflurane across this age [18]. Children were included in the study if a gaseous induction of anaesthesia was required. Examples of the procedures that the children required include MRI scans, squint repair, hernia repair, and orchidopexy. Children were not eligible for inclusion in the study if they received premedication before anaesthetic induction or if they required an intravenous anaesthetic induction. Participants requiring emergency care, having central nervous system disease or developmental delay, were also excluded.

Twelve children were included in the pilot study (age range: 19–143 months). In the main study, subjects were divided into 2 groups: 1) the naïve group (n = 28, age range: 14–153 months) and 2) the local anaesthetic (LA) group (n = 11, age range: 25–83 months) who had topical LA cream applied to the stimulus site.

### 2.2 Anaesthetic procedures

Gaseous induction of anaesthesia was performed following routine anaesthetic practice using sevoflurane (Baxter, Newbury, Berkshire, UK), oxygen, and nitrous oxide. Once stable, a laryngeal mask airway was inserted, nitrous oxide was turned off, and sevoflurane reduced, to achieve an end-tidal concentration of 2.5%. The patients were maintained under sevoflurane monoanaesthesia. The time between the induction of anaesthesia and starting the experimental stimuli was at least 10 minutes (the time for electrode placement), at which point end-tidal nitrous oxide levels were confirmed to be <5% and sevoflurane levels stabilised at an end-tidal concentration of 2.5%, equivalent to 1 minimum alveolar concentration (MAC) of sevoflurane across the participant age range [18]. No recordings were made until these levels were reached and anaesthesia was stable.

A subset of children (n = 11, age range: 28–100 months) had topical local anaesthetic (tetracaine 4% w/w – Ametop Gel; Smith and Nephew Healthcare, London, UK) applied to the dorsum of both hands at least 30 minutes prior to anaesthetic induction. The decision to apply local anaesthetic was made by nursing staff. If nursing staff were not informed by the anaesthetists that children were going to have a gaseous induction, then local anaesthetic was applied in case intravenous anaesthetic induction was required.

### 2.3 Experimental recording techniques

Electrodes were applied to the surface of the skin immediately after anaesthetic induction. Eight EEG recording electrodes (Ambu Neuroline disposable Ag/AgCl cup electrodes; Ambu, Ballerup, Denmark) were positioned on the scalp at Cz, CPz, C3, C4, Fz, FCz, T3, and T4 according to the modified international 10–20 System. Reference and ground electrodes were placed at FPz and the forehead, respectively. EEG conductive paste (Elefix EEG paste, Nihon Kohden, Tokyo, Japan) was used to optimise contact with the scalp. All impedances were kept <5 kΩ by rubbing the skin with EEG preparation gel (NuPrep gel; D.O. Weaver and Co., Aurora, CO, USA) prior to electrode placement. Bipolar electromyography (EMG) electrodes (Ambu Neuroline 700 solid gel surface electrodes) were placed on the biceps brachii to measure flexion withdrawal reflexes. An electrocardiography (ECG) electrode (Ambu Neuroline 700 solid gel surface electrodes) was placed on the left clavicle and referenced to FPz. Electrophysiological activity was acquired with the SynAmps RT 64-channel headbox and amplifiers (Compumedics Neuroscan, Charlotte, NC, USA), with a bandwidth from DC – 400 Hz and a sampling rate of 2 kHz used to acquire the data. CURRYscan7 neuroimaging suite (Compumedics Neuroscan) was used to record the activity. All equipment conformed to the electrical safety standard for medical devices. The electrophysiological recordings were performed in the anaesthetic room and electrodes were removed before the child was moved to the MRI suite or surgical theatre.

### 2.4 Stimulation

**Fig. 2:**
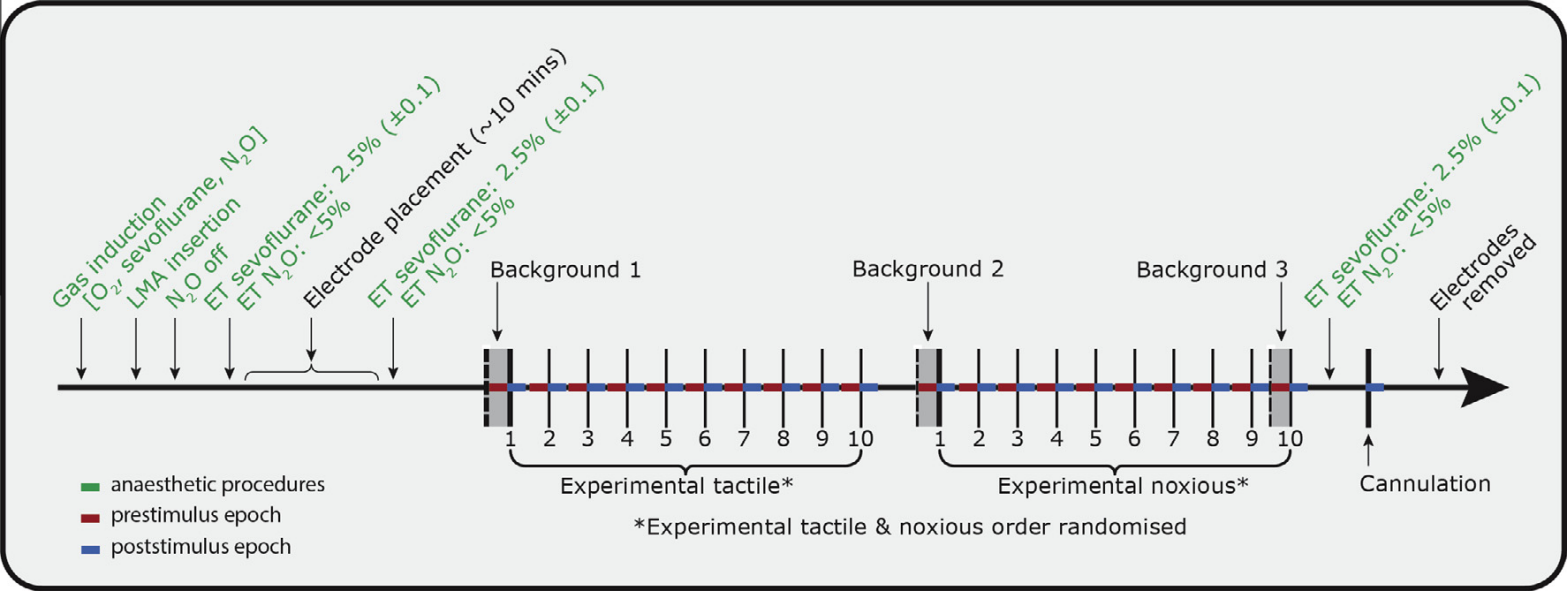
Summary of experimental protocol. Anaesthetic induction was performed following routine practice, with sevoflurane and nitrous oxide. A laryngeal mask airway (LMA) was then inserted and the end-tidal (ET) concentration of sevoflurane was set to 2.5% and nitrous oxide <5%. Following electrode placement, the experimental protocol was carried out, with 2 blocks of experimental stimuli followed by cannulation. The 5-second period before (red) and after (blue) each stimulus were analysed and 3 background periods were taken throughout the recording.

Three types of stimuli were used: experimental tactile, experimental noxious, and clinically required cannulation. A summary of the experimental protocol is given in Fig. 2. Nonnoxious experimental tactile stimuli were applied to the dorsum of the hand using a modified tendon hammer. A train of 10 tactile stimuli were presented to each subject with an interstimulus interval of 11.2 ± 2.5 seconds (mean ± SD). The tactile stimuli were time-locked to the EEG recordings through an event detection interface; the tendon hammer was modified to include an impedance head with a built-in calibrated force transducer (Brüel & Kjær, Naerum, Denmark), which was placed between the rubber tip and handle [36]. Tactile stimuli were event marked with a precision and accuracy of 624 μs and 256 μs, respectively [36].

Experimental noxious (non-skin-breaking) stimuli were applied to the stimulus site using a “PinPrick” stimulator (PinPrick; MRC Systems, Heidelberg, Germany) calibrated to a force of 512 mN. Ten experimental noxious stimuli were presented to each subject with an interstimulus interval of 11.2 ± 1.8 seconds (mean ± SD). The experimental tactile and noxious stimuli were carried out in 2 blocks, but the order in which the stimuli were presented was randomised between subjects. The experimental stimuli were always performed before the routine cannulation. All stimuli, including the cannulation, were performed on the dorsum of the same hand, which was selected by the anaesthetist. There was a slight bias for stimulation to be performed on the left hand (73% of studies). In an attempt to avoid sensitisation, small changes to the stimulation site were selected between experimental stimuli.

**Fig. 3:**
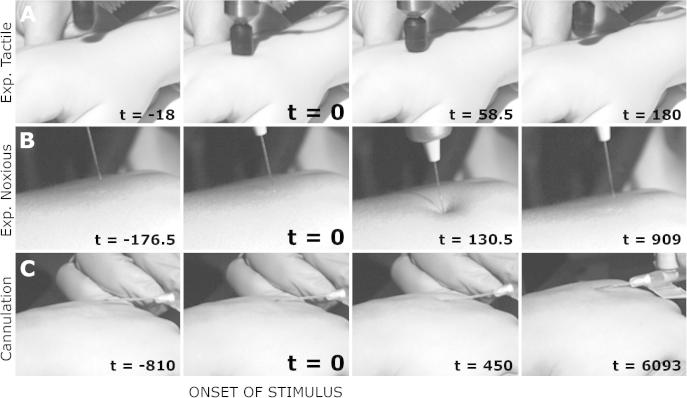
Electroencephalography (EEG) time-locking. Example shots show the video recordings. Experimental tactile stimuli (row A) were time-locked to recordings through an event detection interface. Experimental noxious stimuli (row B) and cannulation (row C) were time-locked to the EEG recording using high-speed video recordings. t = 0 ms refers to the time when the stimuli first came into contact with the skin. The times shown in ms in the frames refer to these particular examples.

Experimental noxious stimuli and cannulation were time-locked to electrophysiological recordings by means of a high-speed camera (Firefly MV, Point Grey Research Inc., Richmond, BC, Canada) that was directly linked to the recordings at the time of acquisition. Video recordings were captured at 220 frames per second, a precision of 9 ms and accuracy of 4.5 ms. The events were marked on the electrophysiological recording after the experiment, and were defined as the point where the stimulus (experimental noxious or cannula) first made contact with the surface of the skin (Fig. 3).

### 2.5 Pilot study

An exploratory pilot study was conducted in 12 children, examining the EEG response to cannulation at Cz and CPz electrodes. Initial analysis demonstrated that vertex-evoked potentials – as would be expected in the awake child – were not observed in response to cannulation, but instead, an increase in delta power was observed at both electrode sites (20.0 ± 13.4% at Cz and 13.5 ± 12.0% at CPz; mean ± SEM). Based on the results from the pilot study, we hypothesised that noxious stimulation in children receiving sevoflurane monoanaesthesia evokes an increase in EEG delta activity, with the primary hypothesis that cannulation would evoke an increase in delta activity at Cz and CPz. This hypothesis was tested in an independent sample of children (n = 39), with our secondary analysis examining whether the response was reduced by application of topical local anaesthetic, whether the increase in delta activity was observed at other electrode sites, and whether the increase in delta activity would also occur in response to experimental noxious and tactile stimulation.

As the pilot study formed part of an exploratory investigation, where aspects of the methodology were refined, there were some differences in the experimental design compared with the main study: there was a slight variation in the end-tidal concentrations of sevoflurane; 3/12 subjects were stimulated on the dorsum of the foot rather than the hand; and some of the children were not assessed to be neurologically normal.

### 2.6 EEG analysis

The EEG trace was segmented into epochs of 5 seconds before and 5 seconds after the stimulus event mark. Ten experimental noxious stimuli were excluded from the analysis because the camera angle did not allow for the precise onset of the stimuli to be determined. In addition, 10 experimental tactile stimuli were excluded due to insufficient recording length. Each experimental stimulus block included a minimum of 8 stimuli suitable for analysis. Cannulation was included in the analysis for all subjects. Each epoch was baseline corrected (to the mean across the whole epoch) to account for a DC shift, a 50-Hz notch filter and low pass filtered at 70 Hz. The power spectrum before and after the stimulus was calculated in MATLAB (version R2013a, MathWorks, Natick, MA, USA) using the fast Fourier transform. Zero-padding was applied to the data to ensure the data length reached the next power of 2. The power in the delta (0–3 Hz), theta (3–8 Hz), alpha (8–12 Hz), and beta (12–30 Hz) frequency bands was defined as the total power within these respective frequency ranges. Power was calculated for each EEG channel separately. To ensure that the delta activity was not driven by a DC shift, we divided the delta power into 2 ranges: infra-slow-delta (0–0.5 Hz) and high-delta (0.5–3 Hz).

To determine whether the background EEG activity was changing throughout the recording as a result of change in anaesthetic depth or drift in the EEG signal, 3 5-second background epochs of EEG were defined: the first epoch was prior to any stimuli, the second was prior to the second train of experimental stimuli, and the third was prior to the last experimental stimulus (Fig. 2).

During the period immediately prior to cannulation, the limb was repositioned and manipulated in preparation for the cannulation. As activity associated with performing cannulation took place during this time period, it was not used as a background period. Therefore, the change in response to cannulation was calculated from the first epoch of background activity (prior to any stimuli – see Fig. 2). This change in activity evoked by cannulation was compared to the change in activity between background epochs 1 and 2.

### 2.7 EMG and ECG analysis

EMG and ECG activity was also segmented into epochs of 5 seconds before and after each stimulus. A high pass filter at 10 Hz, a low pass filter at 500 Hz, and a 50-Hz notch filter were applied to the EMG signals, and they were baseline corrected to the prestimulus mean. The root mean square (RMS) of the signal was calculated in 250-ms windows, and the average RMS value was calculated pre- and poststimulus [7], [28]. The ECG signal was high pass filtered at 12 Hz (as the ECG lead was referenced to a head electrode – FPz – low frequencies associated with EEG signals were therefore removed) and low pass filtered at 40 Hz. The R waves were then determined as the peaks in the signal above a threshold of 3 times the SD and the R wave to R wave (RR) intervals were calculated. Where spurious RR intervals (outside a range of the mean ± SD) were detected, the RR intervals were manually checked and corrected using the raw ECG signal. The average RR interval was calculated pre- and poststimulus.

### 2.8 Statistical analysis

All statistical analysis was implemented in R (The R Project for Statistical Computing). In most cases, the data were nonparametric (determined from Q-Q plots). To examine the differences in band power in all 4 of the frequency bands across the 3 background epochs, the data were normalised by taking the logarithm, and a 3-way multivariate analysis of variance (MANOVA) was performed; with EEG channel, subject age, and background period as factors, and the 4 frequency band powers as variables. The change in delta power between cannulation and background EEG was assessed using a permutation test, with subject age and EEG channel as additional factors, for both the naïve and LA groups. A permutation test was also used to consider the change between prestimulus baseline data, experimental tactile and experimental noxious stimuli, with age, EEG channel, and stimulus number as additional factors. A change in power that was different from 0 was assessed using a one-sample median test with Bonferroni correction for multiple comparisons. All permutation tests were carried out using the R package “lmPerm” with iterations terminated when the estimated SE of the estimated *P*-value was <0.001. For EMG and ECG analysis, the average RMS and heart rate were compared using a Wilcoxon signed-rank test.

## 3 Results

### 3.1 Delta activity is the dominant EEG pattern in anaesthetised children

**Fig. 4:**
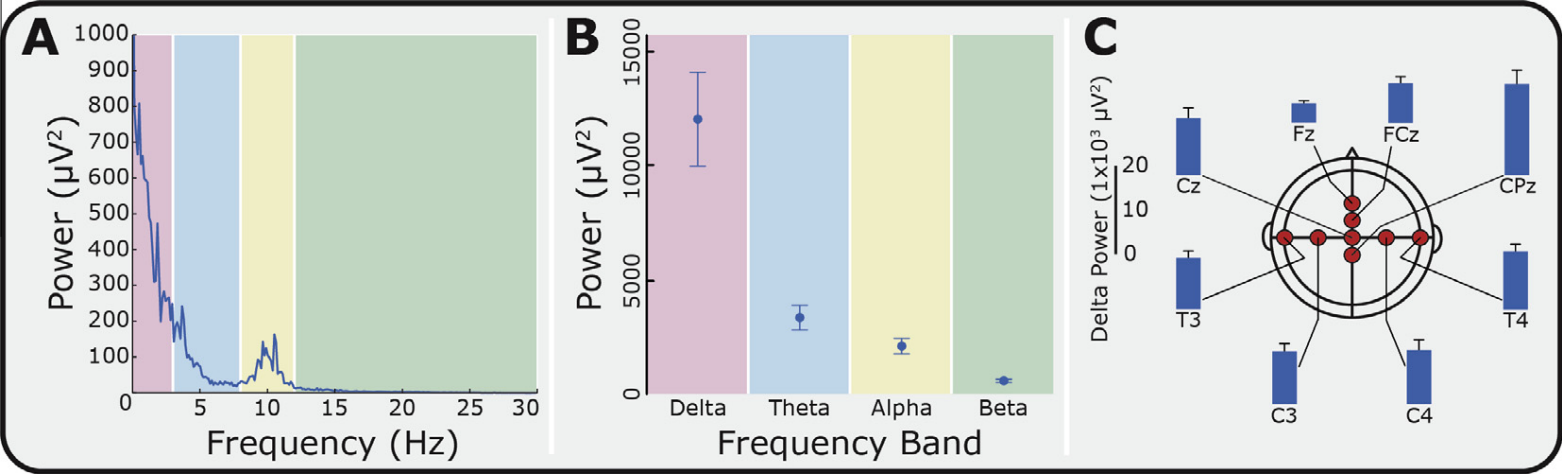
Power-spectra for background electroencephalogram (EEG). (A) Average power spectrum of the background EEG across all channels (Naïve group; n = 28). The spectrum was divided into 4 frequency bands: delta (0–3 Hz, red), theta (3–8 Hz, blue), alpha (8–12 Hz, yellow), and beta (12–30 Hz, green). (B) Comparison of the power in the background EEG in each frequency band. (C) The delta power in the background EEG at each of the EEG channels. Error bars indicate SEM.

The power spectrum of the background EEG, prior to stimulation, averaged across all electrodes and all subjects in the naïve group (n = 28), is shown in Fig. 4A. Consistent with previous reports [6], the majority of activity (62.6 ± 2.8%, mean ± SEM) occurred in the delta band. The percentage of power in the theta, alpha, and beta frequency bands was 18.9 ± 1.5%, 14.3 ± 2.1%, and 4.2 ± 0.5%, respectively (Fig. 4B). The background EEG activity was stable across the whole recording period and no significant differences in power (in any frequency band) occurred across the 3 background epochs (*P* = 0.42; 3-way MANOVA). The level of delta power in the background EEG signal was significantly different between channels (*P* < 0.001), with the highest delta power recorded at CPz (Fig. 4C), but was not significantly different across the subject age range (*P* = 0.11).

### 3.2 Delta power is significantly increased following cannulation, and this response is inhibited by local anaesthetic

**Fig. 5:**
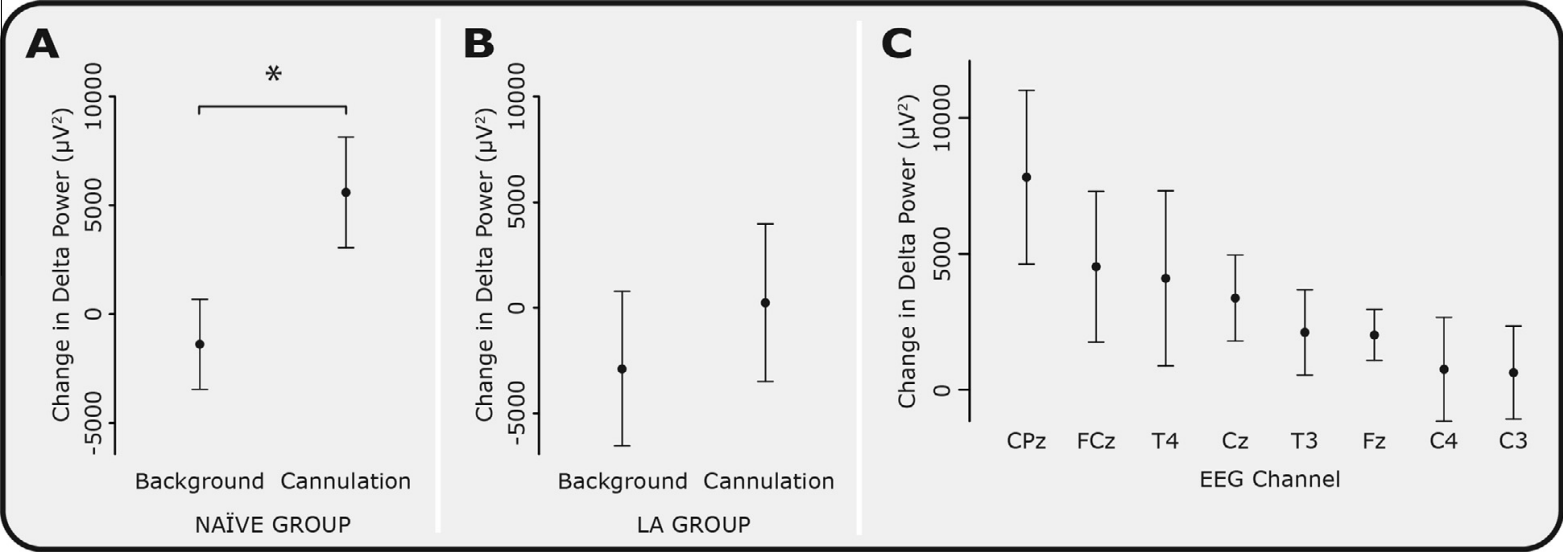
Change in delta band power in response to cannulation. The change in delta band power between cannulation and background activity, compared with the change between 2 background periods at Cz and CPz in (A) the naïve group, and (B) the local anaesthetic (LA) group. Cannulation evoked a significant increase in delta power in the naïve group (^∗^*P* < 0.05), which was not seen in the LA group. (C) Change in delta power between cannulation and background activity at each of the electroencephalogram (EEG) channels (ordered according to mean response). Error bars indicate SEM.

**Supplementary Fig. 1:**
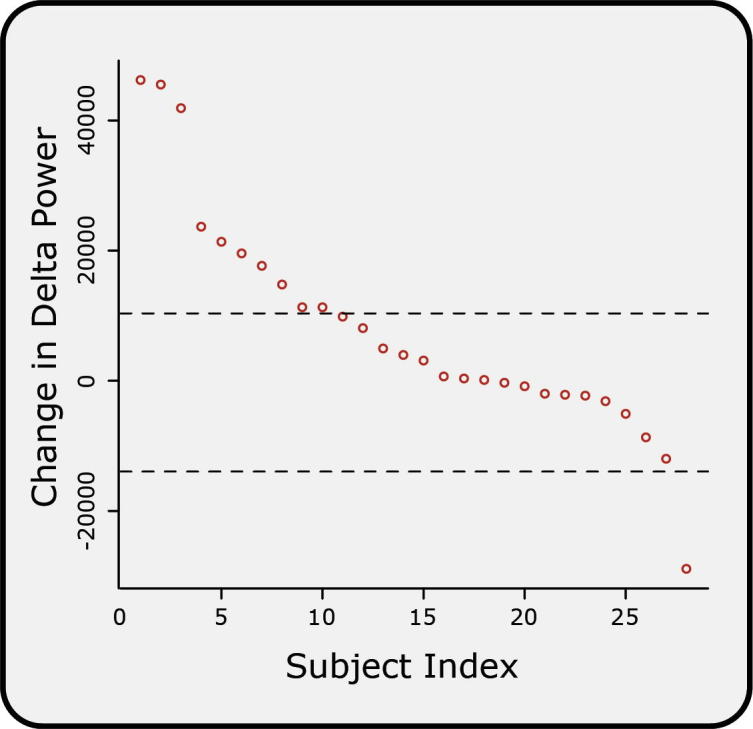
Change in delta power in individual subjects. Individual subjects were ranked according to change in delta power between cannulation and background. Dashed lines indicate the mean ± SD of the change in delta power between the background periods.

The evoked change in delta power post cannulation in the naïve group, at Cz and CPz, was significantly greater than the change in delta power across the background periods (Fig. 5A, *P* = 0.042, permutation test, with no effect of age or channel). Cannulation caused an average 34.2 ± 8.3% increase in the delta power compared with the background activity. Supplementary Fig. 1 shows the change in delta power at CPz evoked by the cannulation for each individual subject, and the mean ± SD of the change across background periods. Thirty-six percent (10/28) of subjects showed an increase in delta activity, above the level of background variability, in response to the cannulation (Supplementary Fig. 1). The application of topical local anaesthetic to the stimulation site prevented the cannulation-induced increase in the delta activity (Fig. 5B). When topical local anaesthetic was applied to the stimulation site (LA group), the mean evoked change in delta activity in response to cannulation was reduced by 95.7% (mean change in delta power post cannulation compared with background EEG in the naïve group = 5598.0 ± 2538.2 μV^2^, and in the LA group = 241.1 ± 3727.4 μV^2^, ±SEM), and was not significantly different from background activity (*P* = 0.30).

**Fig. 6:**
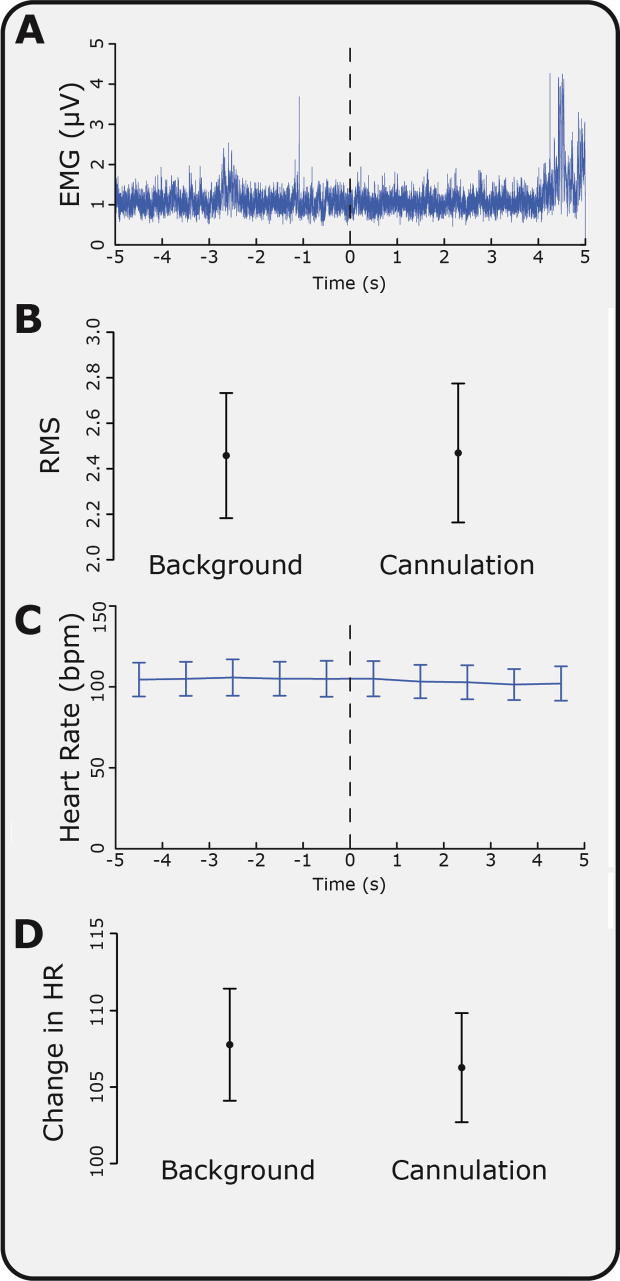
Changes in electromyogram (EMG) and electroencephalogram (ECG) in response to cannulation. (A) Average EMG trace 5 seconds before and after cannulation (across all subjects). (B) Average root mean square (RMS) of EMG activity in response to cannulation and in the background. (C) Average heart rate 5 seconds before and after cannulation (across all subjects). (D) Average heart rate in response to cannulation and in the background. Error bars indicate SEM.

**Fig. 7:**
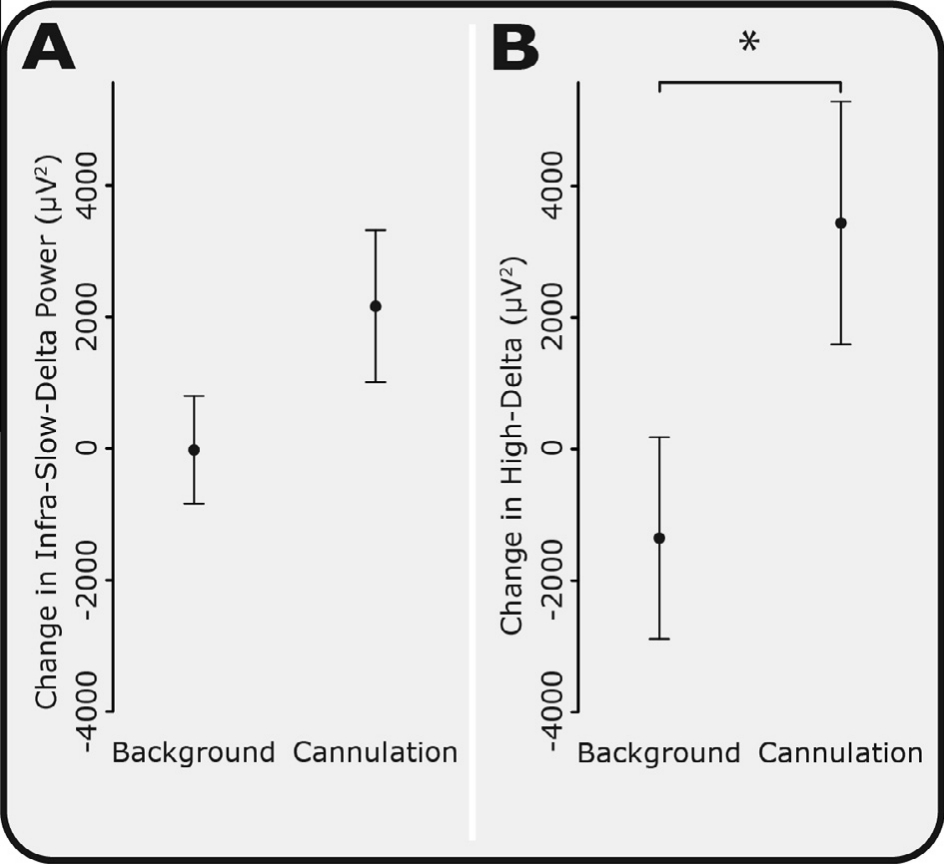
Change in infra-slow and high-delta activity in response to cannulation. To determine whether the delta power response to cannulation was related to infra-slow or higher frequency delta activity, the delta band was split into infra-slow-delta (0–0.5 Hz) and high-delta (0.5–3 Hz) components. (A) Infra-slow-delta increased with cannulation compared with background periods, but this increase was not significant (*P* = 0.13). (B) High-delta significantly increased in response to cannulation (*P* = 0.031). Error bars indicate SEM.

The significant increase in delta power recorded in the EEG in response to cannulation was not associated with movement or a change in heart rate in the same time period. Average heart rate in the background ECG was 107.8 ± 3.7 beats per minute, and post cannulation was 106.3 ± 3.6 beats per minute (mean ± SEM, *P* = 0.09; Fig. 6). There was also no change in the ipsilateral or contralateral EMG activity between background and cannulation (*P* = 0.26 and 0.55, respectively, see Fig. 6). Fig. 5C shows the change in delta power post cannulation compared with background EEG across all recording electrodes. While the largest response was observed at CPz, all electrodes showed an increase in delta power. In order to exclude the possibility that DC artefacts, such as potential changes across the skin, were responsible for the increase in delta activity, power was also examined in the infra-slow-delta band (0–0.5 Hz) and high-delta band (0.5–3 Hz) separately (Fig. 7). There was a significant increase in response to cannulation in the high-delta band (*P* = 0.031) and a nonsignificant increase (*P* = 0.13) in the infra-slow delta band. This suggests that the majority of the evoked change in delta activity was caused by an increase in the high-delta band.

### 3.3 Delta power is increased by experimental noxious and tactile stimuli, and is graded with the intensity of the stimulus

**Fig. 8:**
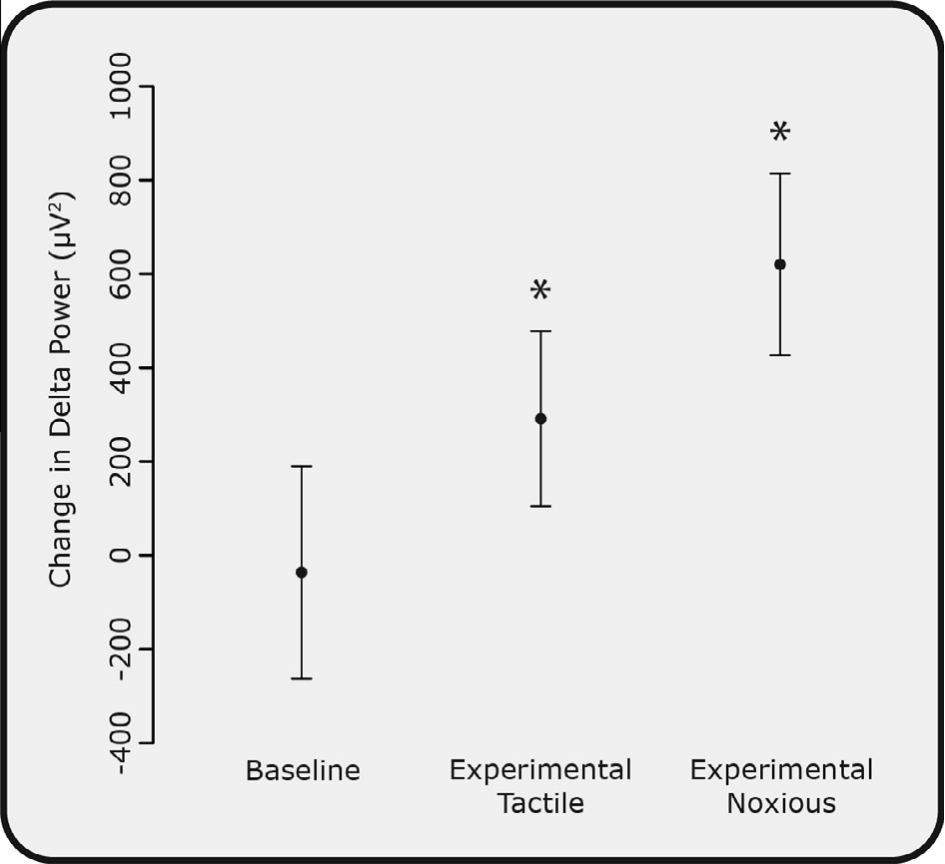
Change in delta power in response to experimental noxious and tactile stimuli. The change in delta band power (poststimulus minus prestimulus) for all experimental tactile and noxious stimulation (^∗^*P* < 0.05, significant difference from 0) compared with the change between sequential prestimulus (baseline) periods. Error bars indicate SEM.

The experimental noxious stimuli (9 ± 1 per subject) applied to the dorsum of the hand caused an average increase in delta activity of 620.4 ± 193.4 μV^2^, which was significantly different from 0 across all electrodes (Fig. 8, *P* = 0.0099, one-sample median test with Bonferroni correction for multiple comparisons). The experimental tactile stimuli (9 ± 1 per subject) also evoked a significant increase in delta power (*P* = 0.012), although the increase was lower (291.4 ± 186.5 μV^2^) than that evoked by the experimental noxious stimuli. Comparison of the innocuous and noxious experimental stimulation, Fig. 8, with the response to cannulation, Fig. 5A, shows a graded increase in delta power with the intensity of the stimulus. There was also no significant difference in the change in delta power with progressive stimulus number (*P* = 0.40, permutation test), indicating that habituation or sensitisation to the stimulus did not occur at this intensity or interstimulus level.

## 4 Discussion

In this study we have used innovative techniques to demonstrate that noxious stimulation results in a significant increase in the EEG delta activity in children receiving sevoflurane monoanaesthesia. This increase in activity was inhibited when topical anaesthetic was applied to the surface of the skin, and cannot be attributed to movement or autonomic responses, as no concomitant changes in either heart rate or spinal reflex withdrawal were recorded in the same time period. Furthermore, there were no differences in the background EEG activity throughout the recording period, implying that no significant drift in anaesthetic depth occurred during the experiment. There was also no effect of age across the age range (1–12 years), suggesting that the stimulus-evoked increase in delta activity is already developed by 1 year of age. The increase in delta activity was graded with intensity of stimulation; both the noxious and tactile stimuli evoked an increase in delta activity, but cannulation evoked a greater increase compared with the experimental noxious and experimental tactile stimuli, and the experimental noxious stimuli evoked a greater increase in delta activity than the experimental tactile stimuli. Given that nociceptive evoked potentials can be recorded from awake infants [30] and adults [15], including in response to single acute noxious stimuli such as a heel lance [30], it is worth noting that no evoked potentials were observed in response to any of the stimuli in this study, including the cannulation.

In EEG recordings in adults, the administration of anaesthetics results in an increase in delta activity compared with awake subjects [22], which is thought to be generated by large networks of synchronously firing cortical neurones. In this study, the administration of sevoflurane monoanaesthesia also resulted in a high level of synchronous firing, with delta activity accounting for 62.6% of the power in the signal. This phenomenon is thought to arise, in part, because thalamic neurones default to their rhythmic burst-firing mode in the absence of ascending excitatory input from arousal nuclei [10], [20], [32]. The extensive connectivity between thalamic and cortical neurones causes large populations of cortical neurones to fire in synchrony, giving rise to delta oscillations [10].

This study provides insight into how the brain in anaesthetised children responds to noxious events. The EEG in an awake subject is predominantly characterised by higher-frequency components and has lower levels of delta activity compared to anaesthetised patients or when subjects are asleep. We have shown that in anaesthetised children, noxious and innocuous events lead to a further increase in delta activity. The mechanisms underlying this phenomenon are not fully understood. Although it is plausible that when a barrage of nociceptive activity arrives at the thalamus of the anaesthetised patient, more thalamic neurons are recruited into the cortical thalamic loop, thus intensifying the cortical oscillations. In an EEG recording, this would be observed as an increase in activity in the delta frequency band [10], which is consistent with the observations in this study.

Similar observations, of noxious stimulation causing increased delta power, have been reported in anaesthetised adults, where a range of clinical events, including surgical incision and laryngoscopy, have been shown to cause an increase in delta activity [3], [12], [16], [31]. The opposite effect (ie, a decrease in delta activity) has also been reported in some studies, with the differences in results attributed to the different surgical stimuli, anaesthetic agents, and dose [2], [3], [26], [31]. In this study, cannulation evoked an increase in delta activity above the expected level of background variability in 36% of the children. The individual differences between subjects cannot be attributed to age, different surgical techniques, or anaesthetic agents or dose. It is possible that the differences reflect the children’s individual variability in terms of their sensitivity to the anaesthetics agents or sensory processing. However, there is a complex interaction between the effects of anaesthesia and noxious stimuli on the generation of delta activity. The children who did not show an increase in delta activity may have received a level of anaesthesia that was sufficient to supress the response. Given recent evidence that suggests that changes in slow wave activity can be used as an individualised biomarker to identify the point when patients transition from awareness to unresponsiveness [22], the importance of investigating the factors that drives this response is clear. As the intensity of stimulation used in this study was low and the evoked increases in delta activity was relatively small, the clinical utility of these measures cannot yet be assessed. Nevertheless, the novel techniques presented in this study provide a methodology whereby standardised levels of anaesthesia and experimental stimulation can be investigated, which will provide a platform in future studies to investigate what drives the inter-subject variability, and thus determine the clinical usefulness for individual patients.

While the increase in delta power observed in this study was small, it is important to note that the noxious stimuli used were minimal compared with surgical events. As this study suggests that the evoked response is graded with the intensity of stimulation, an important next step will be to use these techniques to investigate the effect of more intense surgical events, as well as to investigate a more comprehensive range of the anaesthetic and analgesic strategies currently used in children. The observation that the increase in delta activity observed in this study has also been observed in adults is also of interest, because despite the continuous brain development that occurs throughout childhood and adolescence [11], [14], [25], [34], it appears that the mechanisms involved in the generation of this evoked response are already present from 12 months of age. However, a more extensive developmental study would need to be conducted to establish how this activity is modulated by age.

The high level of delta activity that is a hallmark of the EEG recordings in anaesthetised patients is not unique to anaesthesia. Delta activity is the dominant pattern of background activity in the neonatal brain [1] and is highly prevalent during slow-wave non-rapid eye movement sleep [27]. During slow-wave sleep in adults, noxious stimulation of the muscle and joints has been shown to cause a decrease in delta band power, although no changes in EEG activity were observed following cutaneous noxious stimulation [8]. While there are similarities in the EEG pattern observed in slow-wave sleep and in anaesthetised patients, thought to arise due to the synchronised thalamocortical oscillations, the increase in cortical arousal caused by noxious stimulation in sleep highlights one of the differences between these states. Indeed, in contrast to the anaesthetised patient, it is essential that salient noxious stimuli cause arousal from sleep.

The novel method that we used to time-lock the stimuli to the EEG meant that the precise timing of the sensory input could be recorded. This methodology may provide a more sensitive way of investigating whether current analgesic strategies used by anaesthetists are adequately antinociceptive. Similar methods have been used in other clinical investigations examining nociceptive-evoked brain activity in premature and newborn infants [29], [30]. In this study, the methods were further developed to use a high-frame-rate video camera so that the timing of more complex clinical events, such as a cannulation, could be established. The feasibility of using this approach to event-mark the EEG recording in the anaesthetic room has been confirmed. Furthermore, as video time-locked EEG is often used clinically to relate epileptic brain activity to subtle behavioural manifestations, and most clinical EEG systems can only synchronise the EEG to the video recording at conventional frame rate, that is, with a precision of ∼60 ms, the ability to time-lock EEG to a video recording with a precision of 9 ms, using simple and easily available technology, may be useful in other clinical and experimental settings.

The assessment of adequate analgesia in the anaesthetised patient is inevitably problematic. Any conjecture about pain is inferred from limited surrogate measures such as changes in heart rate and spinal reflex withdrawal activity. However, in the anaesthetic setting, where multiple drugs are used, care must be given to the choice of measures used to assess nociceptive activity; for example, a lack of motor response may be related to the use of muscle relaxants, and so would not in itself imply an adequate level of analgesia. It has been suggested that alternative measures, such as pupillary dilation reflex, may be a more sensitive measure of nociception in anaesthetised children, as clinical doses of sevoflurane, which inhibit autonomic and motor activity, do not inhibit the pupillary dilation reflex [4], [5]. Given that EEG measures cortical activity, which is a fundamental requirement for the experience of pain, perhaps the change in delta power that arises following noxious stimulation could be a useful parameter to consider when assessing if medication administered to anaesthetised patients is antinociceptive. This is particularly interesting in light of our observation that the application of a topical local anaesthetic diminished the evoked increase in delta power caused by cannulation. While the question of whether a nonverbal patient is in pain can never be fully answered, simultaneously recording multiple physiological responses, including cortical activity, and investigating how these responses are altered by different drugs may give the best insight into nociceptive processing [13]. This technique may also provide opportunities for researchers to investigate whether different clinical populations, for example, children who have been born prematurely or who have experienced a high level of early life pain, have differing nociceptive sensitivity during general anaesthesia. Such research may aid clinicians in their provision of adequate analgesia in anaesthetised paediatric patients.

## Conflict of interest statement

The authors declare no competing financial interests.
